# Impact of nickel doping on the optical, magnetic and electrical properties of CMFC ferrite

**DOI:** 10.1038/s41598-026-67441-w

**Published:** 2026-09-10

**Authors:** A. M. Moustafa, A. A. Ward, A. F. Mabied, S. A. Gad

**Affiliations:** 1 Solid State Physics Department, Physics Research Institute, National Research Centre, Dokki, Giza, 12622 Egypt; 2 Microwave Physics and Dielectrics Department, National Research Centre, Dokki, Cairo, 12622 Egypt; 3 Physics Department, Faculty of Science, Galala University, Galala City, 43511 Suez Egypt

**Keywords:** Nickel-substituted CMFC ferrites, Spinel structure, Dielectric properties, Soft magnetic behaviour, High-frequency electronic applications, Materials science, Physics

## Abstract

The multifunctional properties of spinel ferrites can be effectively tailored through Ni substitution, particularly in systems where cation redistribution and electron hopping mechanisms govern the structural, optical, electrical, and magnetic responses. In this work, Cu₀.₇Mg₀.₃₋ₓNiₓFe₁.₇Cr₀.₃O₄ CMFC ferrites (0.05 ≤ x ≤ 0.30) were synthesized and systematically investigated using XRD, SEM, optical and transport properties analysis. Ni incorporation promoted improved lattice ordering and cation redistribution within the spinel structure, leading to modifications in electronic polarizability and charge transport pathways. These changes were reflected in a systematic widening of the optical band gap from 3.2 to 3.7 eV, accompanied by a reduction in the refractive index. Magnetic measurements confirmed the retention of soft ferromagnetic behaviour, although the saturation magnetization gradually decreased from 20.33 to 18.54 emu g⁻¹ due to variations in cation occupancy and superexchange interactions. The saturation magnetization slightly reduced while preserving soft magnetic behavior with low coercivity (44–57 G). The results of the magnetic properties indicated that, the magnetic response for specific applications such as High-Frequency Transformer Cores, Electromagnetic Interference (EMI) Suppression and Magnetic Recording Heads. Dielectric analysis performed over the frequency range 10⁻¹–1 M Hz at 30 °C revealed pronounced low-frequency dispersion associated with Maxwell–Wagner interfacial polarization at low frequency. The combined structural, optical, electrical, and magnetic characteristics indicate that Ni-substituted Cu–Mg ferrites are promising materials for MHz-frequency electronic devices, including high-frequency transformer cores, electromagnetic interference suppression, and magnetic recording components.

## Introduction

The fields of optoelectronics, sensors, magnetic storage devices, and light-induced catalysis are among the technologically relevant materials known as transition metal oxides (TMOs). The energy density of storage devices can be improved by transition metal oxides (TMOs) or mixed transition metal oxides (MTMOs), such as MFe₂O₄ (M = Ni, Co, Cu, and Zn)^1–4^, often referred to as spinel metal ferrites and MCo₂O₄ (M = Cu, Ni, Zn, Mg, and Mn)^1–3,5,6^. The electrochemical behavior of Semiconductor (SCs) was improved by MTMOs’ diverse chemical compositions and numerous metal cation valences^4^. S. Sanskruti S. Kadam et al. investigated CoCr₀.₂Fe₁.₈₋ₓSmₓO₄ spinel ferrite nanoparticles (x = 0.0–0.1) synthesized via the sol–gel auto-combustion method. The samples were characterized using XRD, FTIR, and magnetic measurements. Their results demonstrated that Sm³⁺ substitution induces structural distortion and cation redistribution, enabling effective tuning of magnetic softness and anisotropy. These findings highlight the potential of rare-earth-substituted Co–Cr ferrites as tunable magnetic materials for sensors, recording media, and high-frequency magnetic device applications^7^.

Ferrites are often classified into two groups: hard ferrites and soft ferrites. Hard ferrites have a strong coercivity for magnetization. These materials are used to make permanent magnets used in high-frequency applications, switch-mode power supplies, loudspeakers, washing machines, communication systems, and microwave absorption systems^8,9^. On the other hand, because of their low coercivity, soft ferrites are readily magnetically manipulated. The superior magnetic field conductivity of soft ferrites aided in the creation of high-frequency inductors, transformer cores, and microwave components. These ferrites include undoped copper ferrite (CuFe_2_O_4_) and those doped with other transition elements, which are used in a variety of applications, including a water gas-shift reaction^10^, heterogeneous Fenton catalysts^11^, genomic DNA isolation and purification^12^, liquefied petroleum gas sensors^13^, organic compound oxidation catalysts^14^, and supercapacitor^15^.

A little percentage of Cu²⁺ ions may move from the octahedral B-site to the tetrahedral one in copper ferrite, which is typically a ferrimagnetic spinel with an inverse cation distribution. According to the formula [Cu₁_−y_Fe_y_][Cu_y_Fe₂_−y_]O₄, the degree of inversion, or the inversion parameter y, is the fraction of copper ions at B-sites, where 0 and 1 represent the inverse and normal situations, respectively. The value of y is significantly impacted by the heat-treatment process and the preparation technique. It is well known that the crystallized spinel structure of bulk copper ferrite exists in two phases: the low-temperature body-centered tetragonal and the high-temperature face-centered cubic (Fd3m) defined by a single lattice parameter a = 8.42 Å. Cations are found in two different crystallographic locations for both phases: the octahedral B-site and the tetrahedral A-site. The oxygen octahedra surrounding the B sites are stretched parallel to the c axis and contracted in the ab plane, which is a significant characteristic of the tetragonal phase. FeO₄ tetrahedra have the same dimensions as cubic inverse ferrites and are entirely undistorted despite the significant structure distortion^16^.

The Jahn-Teller (JT) effect, which is defined by the octahedral distortion of the oxygen surrounding the Cu^2+^ions and the elimination of the e.g. orbital degeneracy, is responsible for the structural transition between the two phases^17^. A phase transition from the cubic to the tetragonal phase arises from the Jahn–Teller effect, in which Cu ions migrate from tetrahedral to octahedral sites, inducing lattice distortion and causing either compression or elongation of the crystal lattice, corresponding to c/a < 1 or c/a > 1, respectively^18–20^. Specifically, Cu^2+^ ions preferentially occupy the tetrahedral 8a site when c/a < 1, whereas they stabilize in the octahedral 16 d site when c/a > 1.

Ferrites’ characteristics can generally be improved for new uses by adjusting the particle size and/or substituting the right material. Numerous researchers have documented the use of physical and chemical methods to manufacture copper nano ferrite^21–23^. Ferrites’ characteristics have been tailored using a variety of substitutes, such as La and Cd^24,25^. Ohnishi et al. investigated the necessary temperature for the initiation of bulk crystal deformation from cubic to tetragonal symmetry as well as the critical percentages of copper ions on 16d and 8a sites^26^. In magnetic resonance imaging (MRI), copper ferrite also functions as a temperature-dependent sensor^27^. A promising substance for thermochemical water splitting to produce hydrogen is copper ferrite^28^.

Recent studies have explored the tuning of copper-based ferrites through various synthesis methods and substitutions. For instance, Dhiwahar et al.^29^ utilized a microwave combustion process to synthesize Cu_1−x_Ni_x_Fe_2_O_4_ (0 ≤ x ≤ 0.5) nanoparticles, demonstrating that Ni-incorporation successfully modulates the magnetic saturation (from 17.37 to 31.37 emu/g) and optical band gap (extending from 2.30 to 2.06 eV). While such studies confirm the efficacy of Ni-substitution in single-cubic phase spinel structures, there remains a significant research gap in understanding how simultaneous multi-cation engineering specifically the interplay between Mg^2+^, Ni^2+^, and Cr^3+^ affects superexchange interactions and cation redistribution.

Recent studies have demonstrated that Ni-containing spinel ferrites, such a copper ferrite (CuFe₂O₄) exhibit enhanced electrochemical, dielectric, and magnetic properties, highlighting the important role of Ni incorporation in tailoring their functional performance^2,30–32^. However, these studies have primarily focused on conventional single-substituted ferrite systems. In contrast, the present work explores the combined effects of Mg²⁺/Ni²⁺ and Cr³⁺ substitution on cation redistribution and the resulting structural, optical, magnetic, and electrical properties, providing a broader understanding of multi-cation engineered spinel ferrites. Studies utilizing X-ray diffraction (XRD) confirm the single-phase spinel nature of these compounds, noting that Ni integration typically reduces crystallite size while expanding the host lattice. Spectroscopic analyses via FT-IR and Raman have identified characteristic absorption bands at 465 and 582 cm⁻¹ and localized sublattice disorder, respectively. Furthermore, investigations into electromagnetic and electrochemical behavior reveal frequency-dependent dielectric properties, pseudo-single domain ferromagnetic behavior, and pseudocapacitive trends, collectively highlighting the role of Ni substitution in tailoring CuFe₂O₄ for high-performance lithium-ion batteries^30^. Our goal in this work was to examine how partial substitution of nonmagnetic cations can alter the structural, optical, and transport aspects of copper ferrite powders Cu_0.7_Mg_0.3−x_Ni_x_Fe_1.7_Cr_0.3_O_4_ (where 0.05 ≤ x ≤ 0.3). This study’s primary goals were to synthesize single-phase spinel structure samples using a solid-state process and examine how the amount of Ni doping affected the composition-property relationships within this system.

The novelty of this work lies in the use of a multi-cation engineering strategy, as opposed to the conventional single-ion substitution in spinel ferrites. This approach involves the simultaneous substitution of nickel (Ni²⁺) and magnesium (Mg²⁺) ions at site A, along with the consistent introduction of chromium (Cr³⁺) ions at site B. This multi-cation strategy induces irregular cation redistribution, resulting in unconventional modifications of superexchange interactions and optical bandgap behaviour, thereby distinguishing it from traditional nickel-doped copper ferrites. Specifically, Cu₀.₇Mg₀.₃₋ₓNiₓFe₁.₇Cr₀.₃O₄ (where 0.05 ≤ x ≤ 0.3) compounds were synthesised via a solid-state route to investigate how controlled magnetic substitution influences the interplay between structural, optical, and transport properties within a single-phase spinel framework.

## Experimental

A series of ferrite samples with the composition Cu₀.₇Mg₀.₃₋ₓNiₓFe₁.₇Cr₀.₃O₄ (where 0.05 ≤ x ≤ 0.3) was synthesized using the standard ceramic technique. This process involved high-purity (99.99% purity) precursors from Merck, including CuO, α-Fe₂O₃, Cr₂O₃, MgO, and NiO. The reagents were weighted stoichiometrically according to Eq. (1) and then homogenized in a corundum mortar for 6 h. The resulting powders were calcined in air at 800 °C for 6 h, with a heating rate of 4 °C per minute. After cooling in the furnace to room temperature, the samples were reground and uniaxially pressed into disks at a pressure of 16 tons/cm². Final sintering was conducted in air at 1270 °C for 6 h, also at a rate of 4 °C per minute, followed by controlled cooling to room temperature within the furnace.

1 $$\begin{array}{*{20}{c}} {0.7\left( {{\mathrm{CuO}}} \right)+0.3 - {\mathrm{x}}\left( {{\mathrm{MgO}}} \right)+{\mathrm{x}}\left( {{\mathrm{NiO}}} \right)} \\ {+1.7\left( {{\mathrm{F}}{{\mathrm{e}}_2}{{\mathrm{O}}_3}/2} \right)+0.3\left( {{\mathrm{C}}{{\mathrm{r}}_2}{{\mathrm{O}}_3}/2} \right)} \end{array} \to {\mathrm{C}}{{\mathrm{u}}_{0.7}}{\mathrm{M}}{{\mathrm{g}}_{0.3 - x}}{\mathrm{N}}{{\mathrm{i}}_x}{\mathrm{F}}{{\mathrm{e}}_{1.7}}{\mathrm{C}}{{\mathrm{r}}_{0.3}}{{\mathrm{O}}_4}{\text{ }}\left( {0.05{\text{ }} \leqslant {\text{ }}x{\text{ }} \leqslant {\text{ }}0.30} \right).$$

X-ray diffractograms were obtained at room temperature using a computer-controlled X-ray diffractometer (Diano Corporation, USA) equipped with Fe filtered Co X-ray tube Kα radiation λ = 1.79026 Å, operated at 45 kV and 10 mA. The scanning range was 15–128.5° (2θ), with step size = 0.02° (2θ) and counting time 8 s/step. The scan electron microscope images were obtained using Quartto S thermofisher operating at H. V. (20 kV). FTIR studies were carried out with JASCO 460 PLUS FTIR spectrometer from 400 to 2500 cm^− 1^. A double-beam spectrophotometer, JASCO V-570 model, was used to measure reflectance in the wavelength range of 200–2000 nm. with a precision of 2 nm, to thoroughly describe the reflectance properties. The data exhibited a reflectance spectrum characterized by various characteristics, including peaks and troughs that signify certain electronic and vibrational transitions. The vibrating sample magnetometer (VSM;9600-1LDJ, USA) with a maximum applied field of 20 kOe was used to determine the magnetic characteristics, at room temperature. Dielectric and conductivity measurements were performed using a high-resolution broadband impedance analyzer (Schlumberger Solartron 1260) over the frequency range 0.1 Hz – 1 MHz. To minimize low frequency noise, the sample holder was equipped with electromagnetic shielding. The analyzer was connected to a computer via an IEEE 488 GPIB interface, enabling automated data acquisition through LabVIEW software. Prior to each measurement, calibration was carried out to eliminate stray capacitance. The estimated measurement errors were within 1% for the dielectric constant (ε′) and 3% for the loss tangent (tan δ). Temperature control during measurements was achieved using a precision controller fitted with a Pt 100 sensor. To ensure sample dryness, desiccators containing silica gel were employed, and the specimens were subsequently placed in a measuring cell containing P₂O₅ until the experiments were conducted.

## Results and discussions

### XRD analysis

**Fig. 1:**
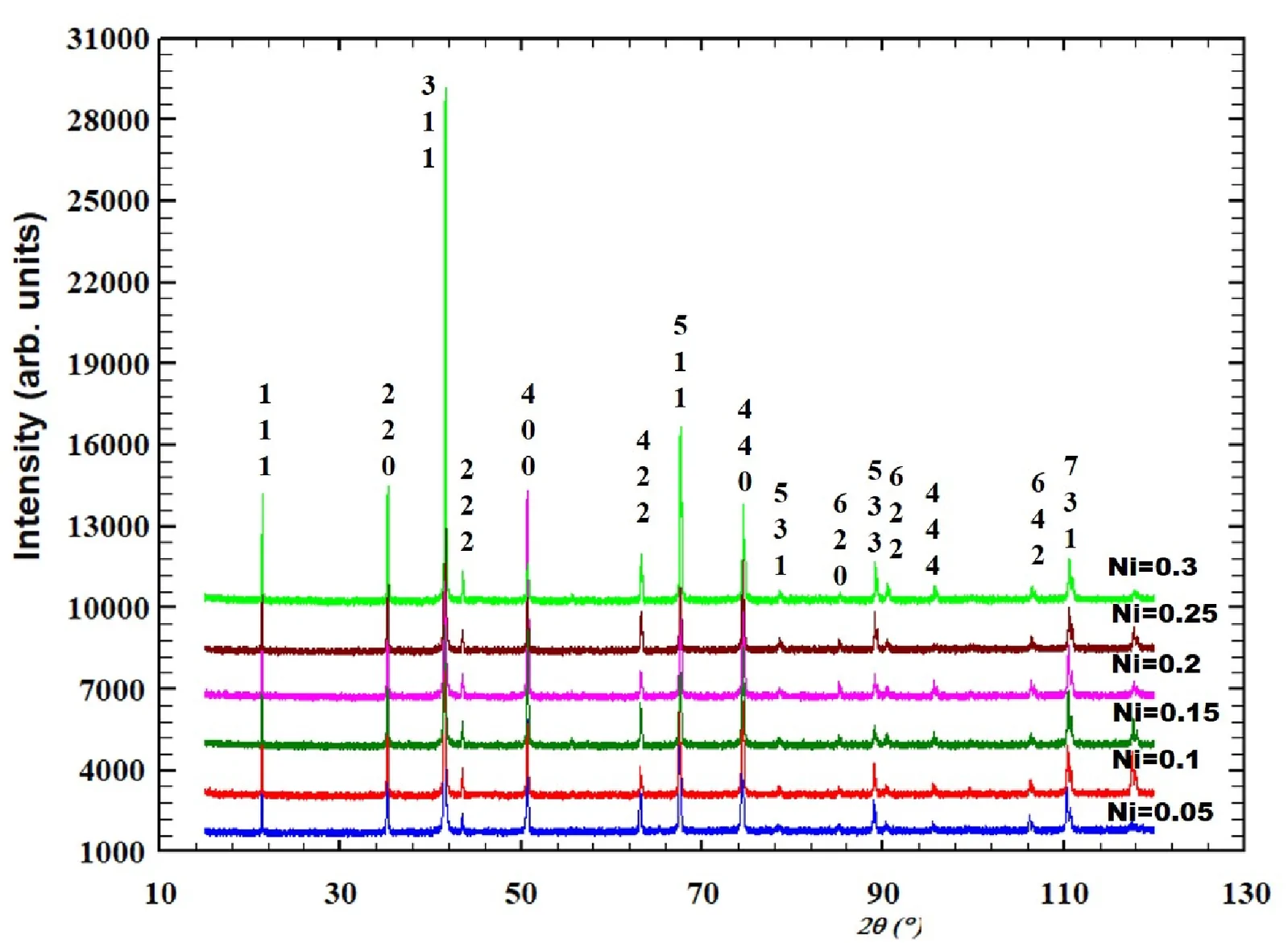
Xray diffraction pattern of Cu_0.7_Mg_0.3−x_Ni_x_Fe_1.7_Cr_0.3_O_4_, (0.05 ≤ x ≤ 0.30).

The X-ray diffraction (XRD) patterns for Cu_0.7_Mg_0.3−x_Ni_x_Fe_1.7_Cr_0.3_O_4_ series, illustrating the effects of varying Ni concentrations, are presented in Fig. 1(a). Phase identification, performed via the X’Pert High Score Plus search-match utility, confirmed the formation of a single-phase cubic spinel structure across all compositions. Detailed parameters regarding the Rietveld refinement applied to these samples are documented in reference^33^. The Rietveld refinement yielded weighted profile residuals (Rwp) of 19.6–24.3%, expected residuals (Rexp) of 12.13–14.10%, and goodness-of-fit (χ²) values ranging from 2.39 to 2.97, indicating satisfactory agreement between the observed and calculated diffraction patterns for laboratory XRD data. In addition, all samples were successfully refined in the cubic spinel structure (space group **Fd-3m**). While these chi^2^ values deviate slightly from the ideal unit value, they represent a high level of agreement for laboratory XRD data, particularly given the structural complexity of the samples. The observed divergence is primarily attributed to microstructural broadening, lattice distortions induced by Ni substitution, and the inherent cation disorder across multiple species (Mg^2+^, Ni^2+^, Fe^3+^, and Cr^3+^) within the spinel lattice. These metrics confirm the statistical reliability of the reported cation distribution.

It should be noted that the proposed cation distribution is inferred from Rietveld refinement together with the complementary FTIR, magnetic, optical, and electrical results, and is consistent with previous reports on Ni-substituted spinel ferrites. Although direct spectroscopic techniques such as Mössbauer spectroscopy or XPS could provide further confirmation, they are beyond the scope of the present study.

For every prepared sample, the Rietveld refinement reveals the centro symmetric structure of space group Fd-3 m. It was discovered that when the Ni content rose, the unit dimensions shrank. Using Eq. (1), as shown in Table 1, the dislocation density (δ)^34^ was computed using the corrected crystallite size data previously published.

2 $$(\delta )\,=\,{\mathrm{1}}/{\mathrm{D}}$$

where the crystallite size is denoted by D. Dislocation density values in the range of 10^− 3^indicate that the ferrite samples’ crystallization has improved and been completed. The decreased dissociation of the particles, where the system lowers its free energy by decreasing the surface area of the particles, may be the cause of the crystallite size increase with increasing Ni concentration^35^.

### SEM analysis

**Fig. 2:**
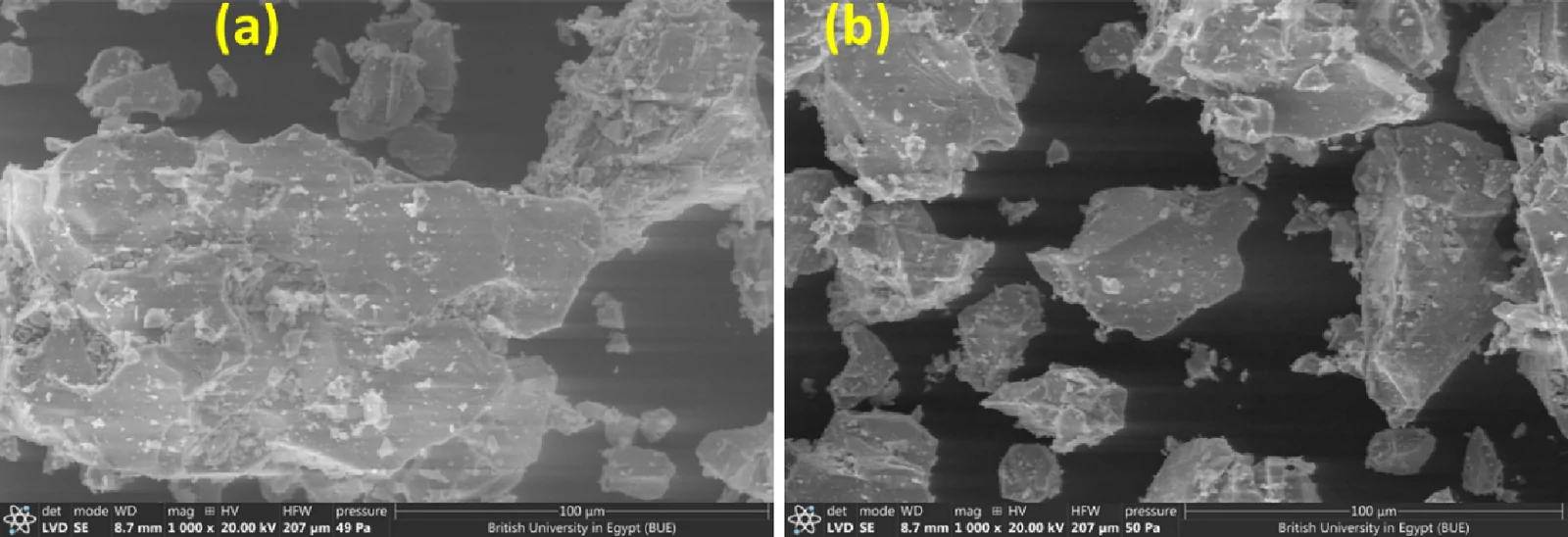
SEM of Cu_0.7_Mg_0.3−x_Ni_x_Fe_1.7_Cr_0.3_O_4_, (0.05 ≤ x ≤ 0.30). (**a**) (Ni = 0.3), (**b**) (Ni = 0.05).

A highly agglomerated polycrystalline microstructure with uneven, faceted grains and neck formation of SEM micrograph of Cu_0.7_Ni0._3_Fe₁._7_Cr_0.3_O₄ is depicted in Fig. 2a. This indicates crystalline development by diffusion-controlled sintering during the high-temperature solid-state process. The observed morphology, in which neighboring particles combine to lower surface energy, is typical of spinel ferrites. Although there are some larger agglomerates, the estimated agglomerated grain size from the SEM image is between 40 and 120 μm. Heterogeneous grain development, which may result from the combined action of several cation substitutions, is indicated by fine particles adorning the surfaces of bigger grains. Specifically, Ni²⁺ encourages particle coalescence and densification, while Cr³⁺ tends to inhibit excessive grain development. Overall, the microstructure is characteristic of solid-state synthesized spinel ferrites, where grain coarsening and agglomeration are anticipated following high-temperature sintering.

Similar morphology can be seen in Fig. 2b, which shows irregularly faceted grains with an estimated agglomerated grain size of 20–90 μm. Despite significant particle coalescence, the existence of intergranular pores and gaps suggests that densification is still not complete. The comparatively reduced grain size in comparison to Fig. 2a indicates that the Mg²⁺/Ni²⁺ substitution influences the kinetics of grain growth, with Ni²⁺ promoting diffusion and local densification and Mg²⁺ serving as a grain-growth inhibitor. Through grain-boundary and porosity effects, these heterogeneous microstructures are frequently described for substituted spinel ferrites and are crucial in defining their magnetic and electrical properties.

### FTIR analysis

**Fig. 3:**
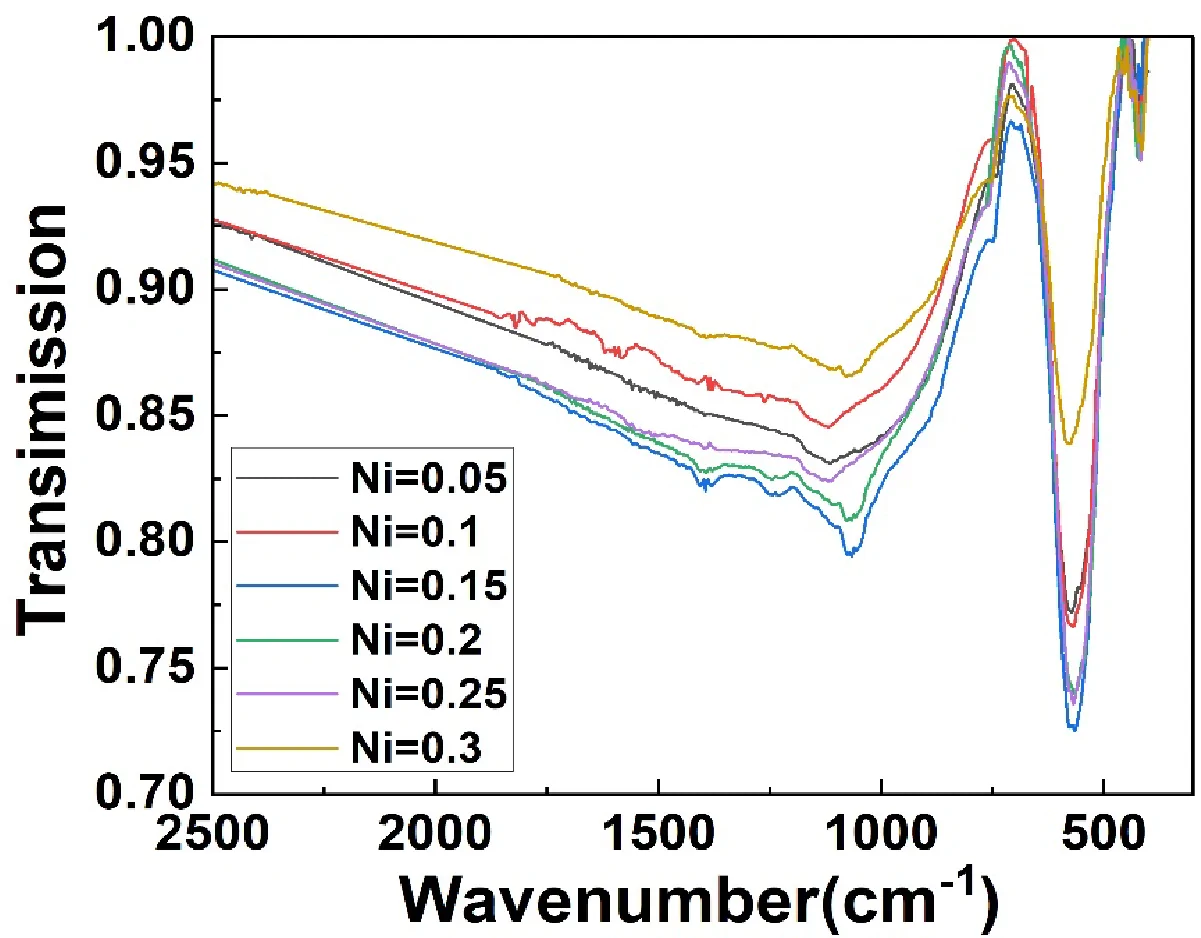
Dependence of Transmission on the wavenumber of Cu_0.7_Mg_0.3−x_Ni_x_Fe_1.7_Cr_0.3_O_4_, (0.05 ≤ x ≤ 0.30).

This measurement method is effective for identifying compounds and analyzing their structures since it uses infrared spectroscopy. The most widely used example is Fourier transform infrared spectroscopy (FTIR), which analyses a material’s chemical structure using infrared light. This is explained by monitoring changes in absorbance, which are caused by the compounds’ vibrating molecules absorbing infrared light at a specific wavelength. Additionally, because of differences in the structural characteristics brought about by the dynamic constants of the bonds and the conversion mass of the atoms connected at both ends, each sample absorbs infrared light at a distinct wavelength. Consequently, FTIR offers exceptional accuracy and precision in determining molecule structures. Figures 3 shows the present infrared spectra of Cu_0.7_Mg_0.3−x_Ni_x_Fe_1.7_Cr_0.3_O_4_ (where 0.05 ≤ x ≤ 0.3). It is evident that the spinel structure is linked to the two basic absorption bands, ν_1_ and ν_2_. The stretching vibrations of the cations at the bonds of the tetrahedral site (A) are associated with the higher frequency band ν1 in the range 565.36–576.25 cm^− 1^. Because the octahedral site’s bond length is longer than that of the tetrahedral site and the frequency is inversely proportional to the bond length^36^, the lower frequency, ν_2_ band in the range 421.337–439.79 cm^− 1^ corresponds to the bending vibrations of the octahedral site’s (B), Table 2 clarify a comparison with other references^37–39^. The band position and shape are greatly influenced by a number of uncontrollable parameters, including synthesis conditions, annealing temperature, and others, in addition to the sample’s chemical composition^40^. Ni^2+^ doping causes a noticeable broadening of the higher frequency band associated with the distribution of Fe at both A&B sites; Patil et al. previously achieved the same behavior in cadmium-doped cobalt ferrite^41^. Smaller intrinsic crystal field splitting in tetrahedral sites, moderate splitting in octahedral sites influenced by ligand field strengths, distortions and disorder that broaden and dampen distinct band splitting in the IR spectra, and the Jahn-Teller effect are the main causes of the observed “weak splitting” of the FTIR bands for octahedral and tetrahedral complexes^40,42,43^. Together, these elements result in a weaker and less noticeable splitting of FTIR bands associated with octahedral and tetrahedral complexes. Peaks in the 1067–1118 cm^− 1^range are linked to binding formation that occurs during copper ferrite production^44^.

### Optical properties

**Fig. 4:**
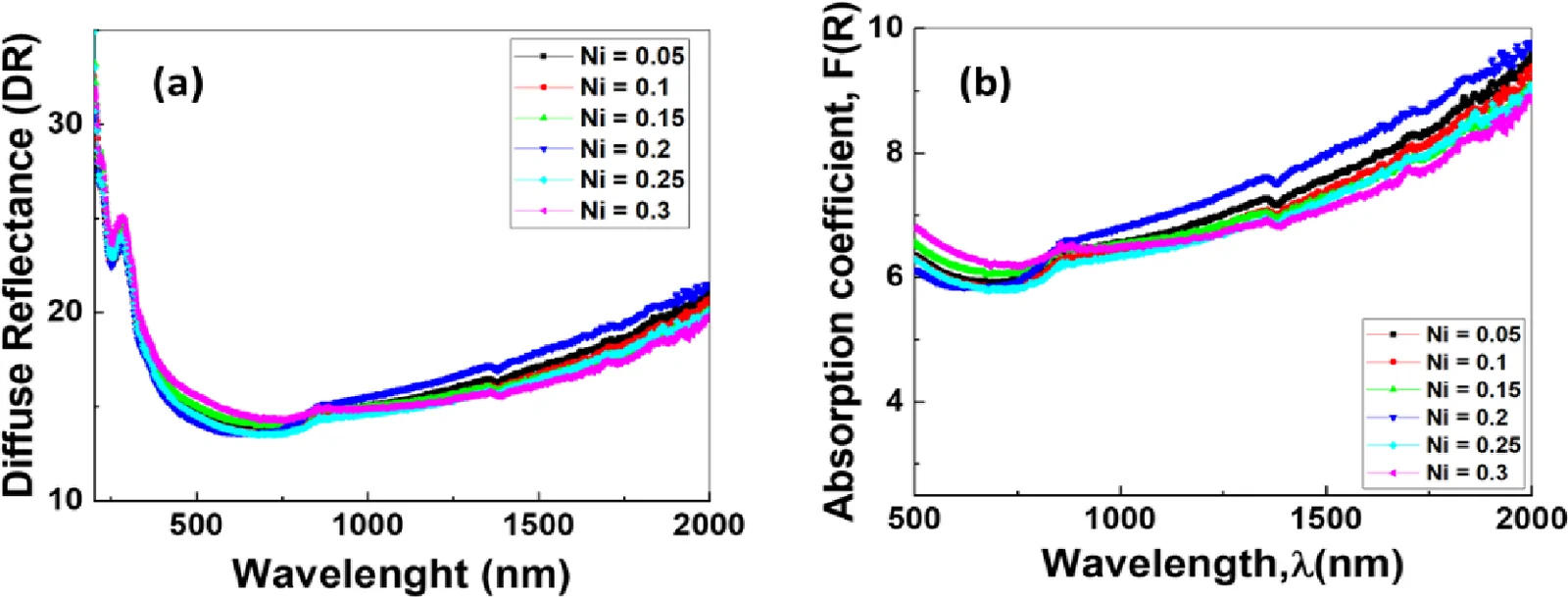
(**a**) Diffuse reflectance vs. wavelength. (**b**): Absorption coefficient vs. wavelength of Cu_0.7_Mg_0.3−x_Ni_x_Fe_1.7_Cr_0.3_O_4_, (0.05 ≤ x ≤ 0.30).

Figure 4a displays the Cu_0.7_Mg_0.3−x_Ni_x_Fe_1.7_Cr_0.3_O_4_(where 0.05 ≤ x ≤ 0.3), samples’ UV-Vis’s reflectance spectra as a function of wavelength in the 200–2000 nm range. It is evident that as the wavelength increased, it also increased Diffuse reflectance (DR). DR generally increases with wavelength (λ) due to a combination of lower absorption and enhanced scattering at longer wavelengths^45,46^. The observed gradual absorption edge is attributed to defect-induced localized states (Urbach tail), structural disorder, and grain boundary effects, which are typical in polycrystalline ferrites synthesized via the solid-state reaction method.

3 $$\:F\left(R\right)=\frac{{(1-R)}^{2}}{2R}$$

The absorption coefficient is represented by F(R), and the reflectance by R. The absorption coefficients F(R) for Cu_0.7_Mg_0.3−x_Ni_x_Fe_1.7_Cr_0.3_O_4_are displayed in the Fig. 4b as a function of wavelength. We can see from the figure that F(R) rises as λ increases. The Tauc relation can be used to calculate the band gap energy of samples^47–49^.

4 $$\:F\left(R\right)=\frac{B{\left(h\nu\:-{E}_{g}\right)}^{n}}{h\nu\:}=\alpha\:$$

**Fig. 5:**
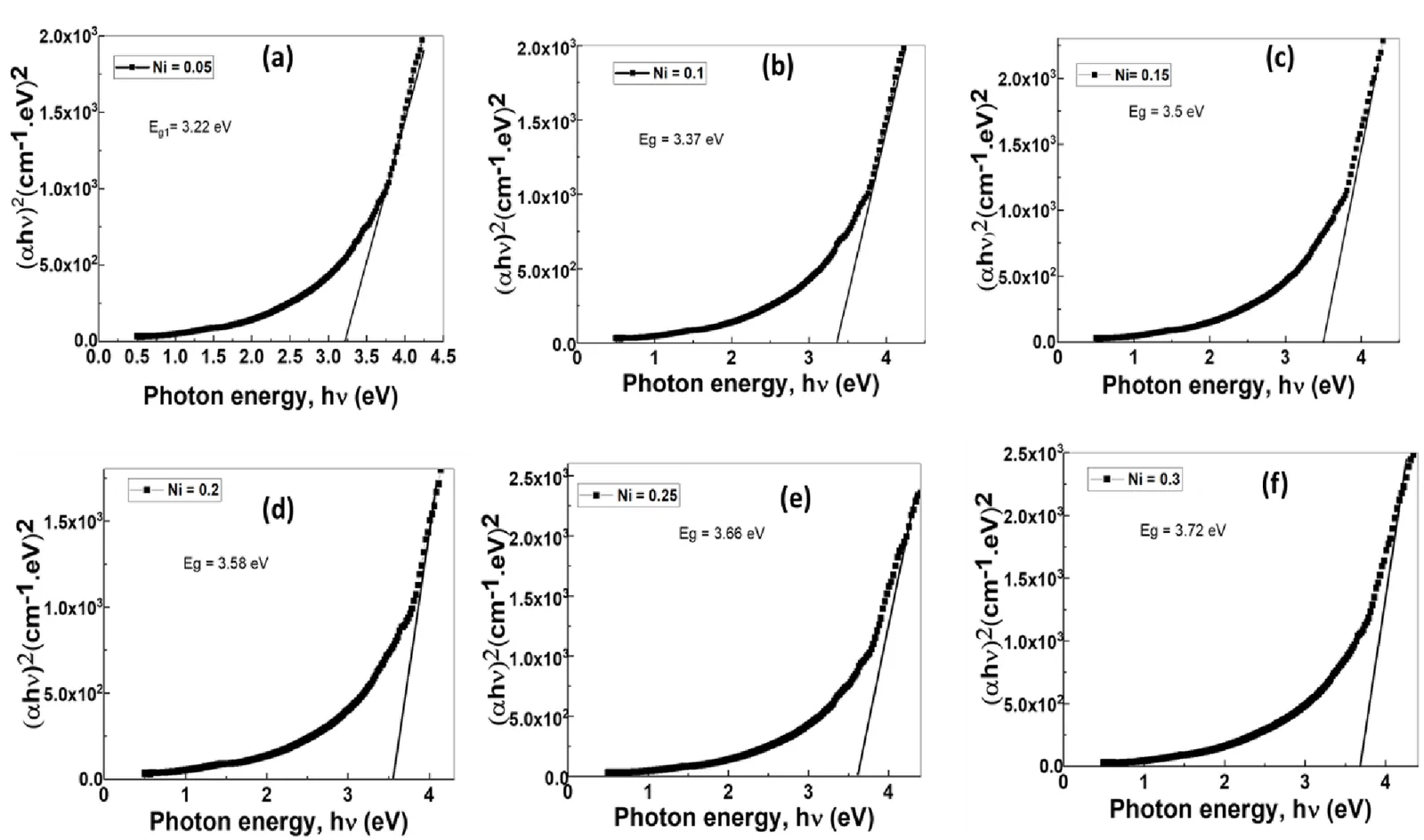
(**a**–**f**): The relation between (αhν)^2^ vs. hν of Cu_0.7_Mg_0.3−_ₓNi_x_Fe_1.7_Cr_0.3_O₄(0.05 ≤ x ≤ 0.30).

Where n is determined by the type of electronic transition, hυ is the photon energy, E_g_ is the optical band gap, and α is the absorption coefficient. Tauc plots were created using several values of n that corresponded to both direct and indirect transitions in order to determine the best suitable transition type. Plotting and extrapolating a graph between [F(R) hυ]^n^ and hυ will therefore yield E_g_ values corresponding to the various Ni concentrations. It was discovered that *n*= 1/2 (allowed direct transition) produced the highest linearity in the high-absorption area, indicating that direct is the predominant optical transition in these ferrites. Where the complex electronic structure including Fe–O, the impact of cation disorder, and lattice distortions frequently favor direct transitions. The linear portion of the curve in Fig. 5a–f was used to calculate these values, which came out to be 3.22, 3.37, 3.5, 3.58, 3.66 and 3.72 eV. This increase could be due to electronegativity brought about by the substitution of Ni atoms for Mg atoms^50^.

**Fig. 6:**
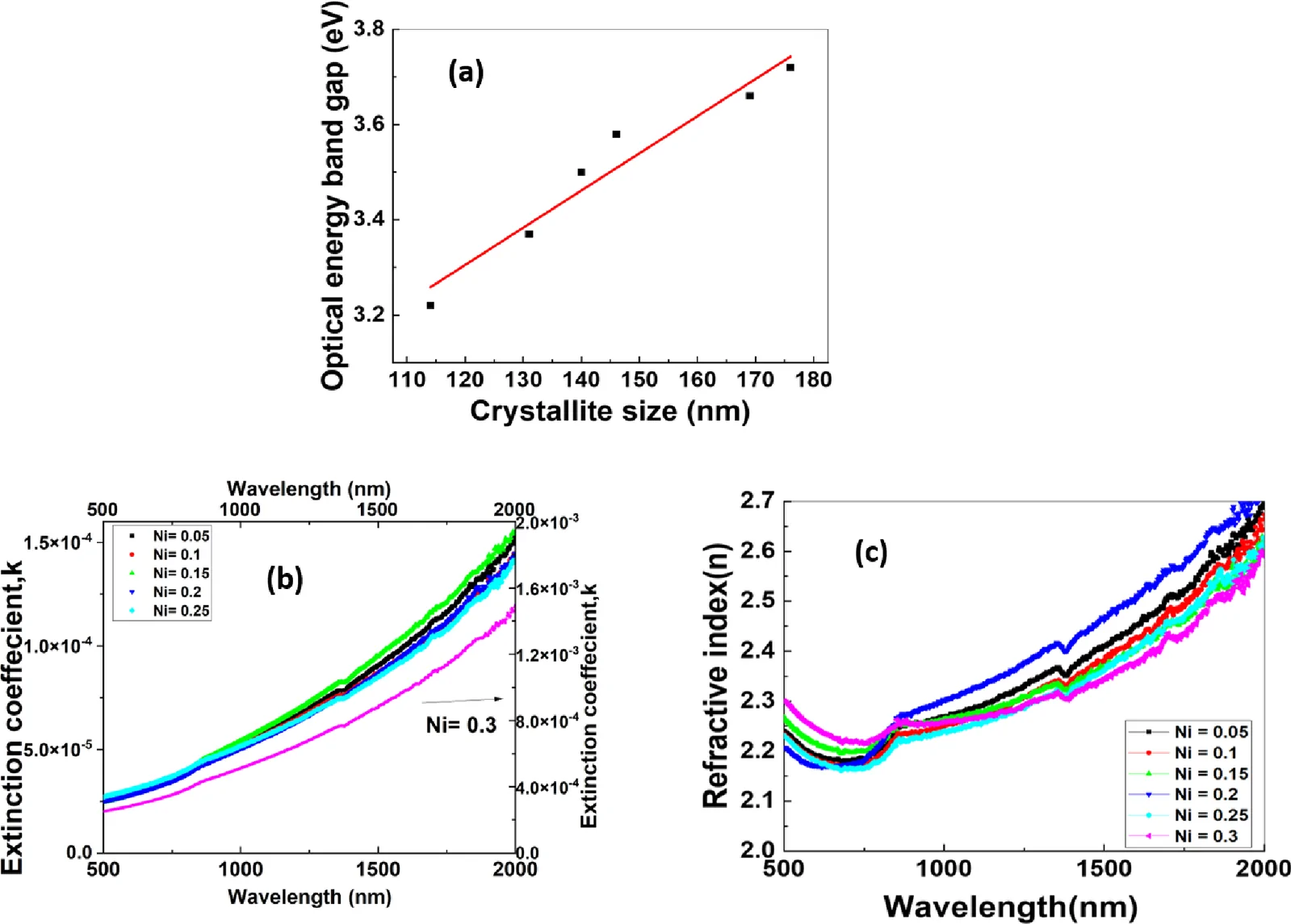
(**a**) Relation between crystallite size (nm) and optical energy band gap (eV). (**b**) Extinction coefficient against wavelength. (**c**): Refractive index vs. wavelength of Cu_0.7_Mg_0.3−x_Ni_x_Fe_1.7_Cr_0.3_O₄ (0.05 ≤ x ≤ 0.30).

The optical band gap (E_g_) of the Ni-substituted ferrite samples exhibits a consistent blue shift from 3.22 to 3.72 eV, indicating band gap widening, which correlates positively with the increase in crystallite size determined by X-ray diffraction from 114 to 176 nm, Table 1. This relationship is illustrated in Fig. 6a. The expansion of the band gap is primarily attributed to enhanced crystallinity and a concomitant reduction in structural disorder and defect-mediated sub-band states. Mechanistically, this trend is driven by cation redistribution within the spinel lattice^51^. As Ni²⁺ ions (0.69 Å) preferentially occupy octahedral (B) sites, they displace Mg²⁺ ions (0.71 Å) and Fe³⁺ ions toward the tetrahedral (A) sites. This migration necessitates the formation of vacancies to maintain charge neutrality when substituting lower-valence cations^52^. The resulting lattice compression enhances crystal-field splitting, leading to an upward shift of the valence band maximum, which is primarily derived from O 2p orbitals hybridized with transition-metal d states, thereby contributing to band-gap widening. Accordingly, the observed increase in Eg can be attributed to the combined effects of Ni-induced modification of Fe–O electronic interactions, reduced defect density, and improved long-range structural ordering and crystallinity^53^. Lattice strain brought on by Ni substitution is anticipated to contribute in addition to cation redistribution. Ni²⁺ ions can cause local distortions in the crystal lattice because they have a different ionic radius than Mg²⁺ ions. These aberrations could have an impact on electron localization and bandwidth, which would explain the observed rise in the optical band gap.

Quantum confinement is unlikely to contribute to the observed band-gap widening because the crystallite sizes (114–176 nm) are far larger than the characteristic dimensions required for quantum size effects in spinel ferrites. Furthermore, the concurrent increase in crystallite size and band gap is inconsistent with quantum confinement. Therefore, the increase in band gap is attributed to Ni-induced cation redistribution, improved crystallinity, reduced defect density, and the associated modification of the electronic structure.

For many devices, it is essential to have a precise understanding of the refractive index (n) and its frequency dependency. The extinction coefficient () is the proportion of light lost due to scattering and absorption per unit distance of the penetrating medium. It is possible to estimate n and k using the following relations^54^:

5 $$\:k=\frac{\alpha\:\lambda\:}{4\pi\:}$$

6 $$\:R=\frac{{(n-1)}^{2}+{k}^{2}}{{(n+1)}^{2}+{k}^{2}}$$

7 $$\:n=\frac{(1+R)}{(1-R)}+\sqrt{\frac{4R}{{(1-R)}^{2}}}-{k}^{2}$$

Regarding Cu_0.7_Mg_0.3−x_Ni_x_Fe_1.7_Cr_0.3_O_4_ (where 0.05 ≤ x ≤ 0.3), the Fig. 6b, c show how k and refractive index (n) relate to λ. Evaluating the refractive indices of optical materials is essential for applications in integrated optics systems, where the refractive index is a crucial design parameter for switches, filters, modulation, and other devices. It is clear that when λ rises, a change in the absorption coefficient causes k to rise as well. Furthermore, the increase in k with λ can be connected to the amount of light absorbed in the samples at higher λ. It should be noted that when the Ni content increases within the wavelength, the values of “n” decrease. The alterations in refractive index in the Cu_0.7_Mg_0.3−x_Ni_x_Fe_1.7_Cr_0.3_O_4_(where 0.05 ≤ x ≤ 0.3) are caused by changes in the polarizability of the component ions^54^.

### Magnetic properties

**Fig. 7:**
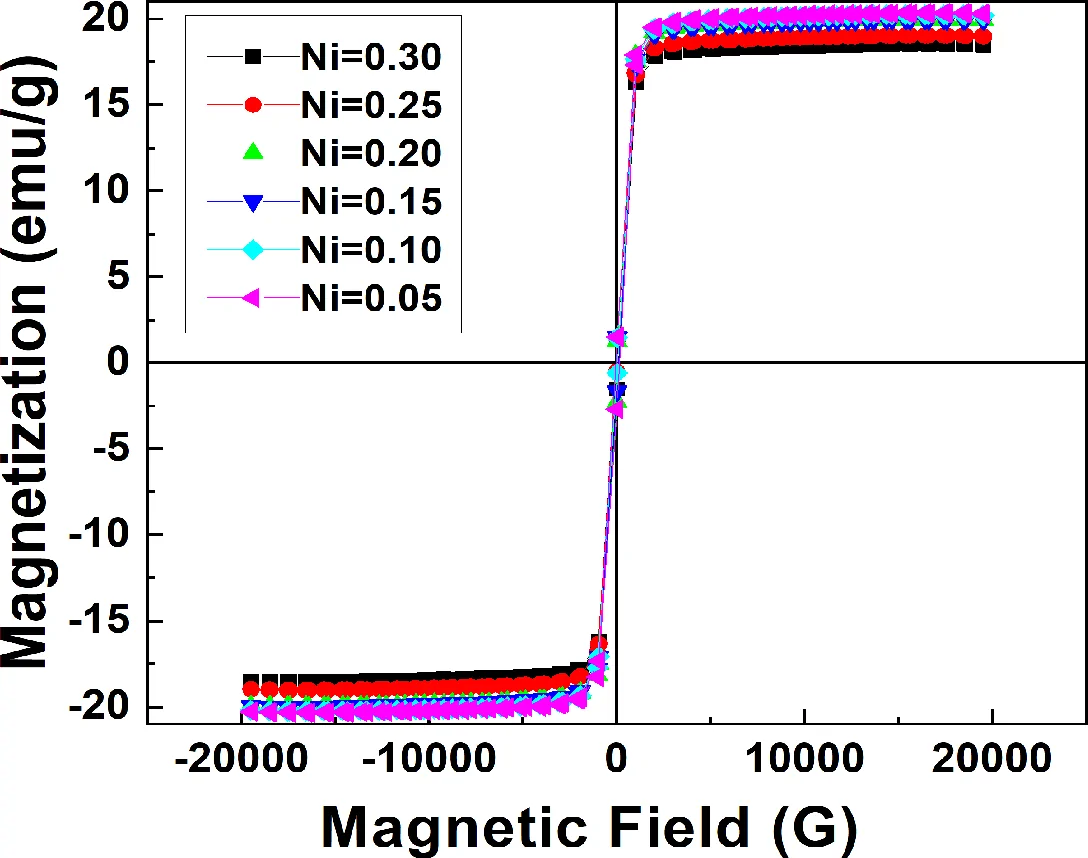
Hysteresis loop for Cu_0.7_Mg_0.3−x_Ni_x_Fe_1.7_Cr_0.3_O₄ (0.05 ≤ x ≤ 0.30).

Figure 7 displays the hysteresis loops of the Cu_0.7_Mg_0.3−x_Ni_x_Fe_1.7_Cr_0.3_O_4_ (where 0.05 ≤ x ≤ 0.3) using VSM (where 0.0 ≤ x ≤ 0.3). These charts illustrate how the applied magnetic field H affects the magnetization M. The fields of application reach 2 × 10^³^ G. The observed M–H hysteresis loops confirm that the prepared samples are soft ferrite materials, characterized by low coercivity and easy magnetization reversal. These ferrites soft magnetic nature is demonstrated by their small hysteresis loops. These curves were used to calculate the coercive field (H_c_), remanent magnetization (M_r_), and saturation magnetization (M_s_). The results are shown in Table 3. Up to 2000 G, the sample’s magnetization grows linearly with an increase in the applied magnetic field; after this, it reaches its maximum values and reaches saturation. This chart makes it evident that as the Ni content rises, saturation magnetization falls. Neel’s notion of sublattice^55,56^ can be used to describe this behavior; the net magnetization will be

8 $$|{\mathrm{M}}|{\text{ }} = {\text{ MB}} - {\text{ MA}}$$

where A and B sites’ magnetizations are denoted by MA and MB, respectively.

The distribution of Fe^3+^ ions between sites A and B determines the magnetization in each sample. According to previously reported Rietveld refinement studies^33^, In spinel ferrites, Cu²⁺ and Ni²⁺ ions preferentially occupy the octahedral (B) sites, whereas Fe³⁺ ions are distributed over both tetrahedral (A) and octahedral (B) sites. The substitution of Mg²⁺ by Ni²⁺ modifies the cation distribution and A–B superexchange interactions, thereby influencing the lattice parameters, crystallite size, and the resulting physical properties. The cation distribution for the samples was as follows:

$$\left[ {{\mathrm{C}}{{\mathrm{u}}_{0.{\mathrm{7}}}}{\mathrm{M}}{{\mathrm{g}}_{0.{\mathrm{25}}}}{\mathrm{F}}{{\mathrm{e}}_{0.0{\mathrm{5}}}}} \right]{\text{A }}\left[ {{\mathrm{N}}{{\mathrm{i}}_{0.0{\mathrm{5}}}}{\mathrm{F}}{{\mathrm{e}}_{{\mathrm{1}}.{\mathrm{65}}}}{\mathrm{C}}{{\mathrm{r}}_{0.{\mathrm{3}}}}} \right]{\mathrm{B}}{{\mathrm{O}}_{\mathrm{4}}}$$

$$\left[ {{\mathrm{M}}{{\mathrm{g}}_{0.{\mathrm{2}}}}{\mathrm{C}}{{\mathrm{u}}_{0.{\mathrm{6228}}}}{\mathrm{F}}{{\mathrm{e}}_{0.{\mathrm{1772}}}}} \right]{\text{A }}\left[ {{\mathrm{C}}{{\mathrm{u}}_{0.0{\mathrm{772}}}}{\mathrm{N}}{{\mathrm{i}}_{0.{\mathrm{1}}0}}{\mathrm{F}}{{\mathrm{e}}_{{\mathrm{1}}.{\mathrm{5228}}}}{\mathrm{C}}{{\mathrm{r}}_{0.{\mathrm{3}}}}} \right]{\mathrm{B}}{{\mathrm{O}}_{\mathrm{4}}}$$

$$\left[ {{\mathrm{C}}{{\mathrm{u}}_{0.{\mathrm{31}}0{\mathrm{5}}}}{\mathrm{M}}{{\mathrm{g}}_{0.{\mathrm{15}}}}{\mathrm{F}}{{\mathrm{e}}_{0.{\mathrm{5395}}}}} \right]{\text{A }}\left[ {{\mathrm{C}}{{\mathrm{u}}_{0.{\mathrm{3895}}}}{\mathrm{N}}{{\mathrm{i}}_{0.{\mathrm{15}}}}{\mathrm{F}}{{\mathrm{e}}_{{\mathrm{1}}.{\mathrm{16}}0{\mathrm{5}}}}{\mathrm{C}}{{\mathrm{r}}_{0.{\mathrm{3}}}}} \right]{\mathrm{B}}{{\mathrm{O}}_{\mathrm{4}}}$$

$$\left[ {{\mathrm{M}}{{\mathrm{g}}_{0.{\mathrm{1}}}}{\mathrm{F}}{{\mathrm{e}}_{0.{\mathrm{9}}0}}} \right]{\text{A }}\left[ {{\mathrm{C}}{{\mathrm{u}}_{0.{\mathrm{7}}}}{\mathrm{N}}{{\mathrm{i}}_{0.{\mathrm{2}}0}}{\mathrm{F}}{{\mathrm{e}}_{0.{\mathrm{8}}}}{\mathrm{C}}{{\mathrm{r}}_{0.{\mathrm{3}}}}} \right]{\mathrm{B}}{{\mathrm{O}}_{\mathrm{4}}}$$

$$\left[ {{\mathrm{M}}{{\mathrm{g}}_{0.0{\mathrm{5}}}}{\mathrm{F}}{{\mathrm{e}}_{0.{\mathrm{95}}}}} \right]{\text{A }}\left[ {{\mathrm{C}}{{\mathrm{u}}_{0.{\mathrm{7}}}}{\mathrm{N}}{{\mathrm{i}}_{0.{\mathrm{25}}}}{\mathrm{F}}{{\mathrm{e}}_{0.{\mathrm{75}}}}{\mathrm{C}}{{\mathrm{r}}_{0.{\mathrm{3}}}}} \right]{\mathrm{B}}{{\mathrm{O}}_{\mathrm{4}}}$$

$$\left[ {{\mathrm{F}}{{\mathrm{e}}_{{\mathrm{1}}.0}}} \right]{\mathrm{A}}\left[ {{\mathrm{C}}{{\mathrm{u}}_{0.{\mathrm{7}}}}{\mathrm{N}}{{\mathrm{i}}_{0.{\mathrm{3}}}}{\mathrm{F}}{{\mathrm{e}}_{0.{\mathrm{7}}}}{\mathrm{C}}{{\mathrm{r}}_{0.{\mathrm{3}}}}} \right]{\mathrm{B}}{{\mathrm{O}}_{\mathrm{4}}}.$$

Cu^2+^ ions, which have a lower magnetic moment of 1 µB, have the highest occupancy at the tetrahedral site at the lowest Ni concentration (Ni = 0.05), while Fe^3+^ ions, which have a greater magnetic moment of 5 µB, have the lowest occupancy at the octahedral site. Additionally, other magnetic ions like Cr^3+^ (3 µB) and Ni^2+^ (2 µB)^57^ occupy the octahedral position. which arises from the difference between the B and A sublattices, causes high values of the effective magnetic moment (8.3 µB) at Ni = 0.05, which in turn causes increased saturation magnetization. The cations in the sublattices rearrange when the Ni content rises, as Ni has a significant predilection for the B-site. In this instance, more Cu ions with a relative site preference for the B-site will increase its presence there at the expense of Fe^3+^ions, which migrate to the A-site and increase the A-site’s magnetic moment while decreasing the B-site’s. As a result, the system’s net magnetic moment will decrease = 0.7 µB at Ni = 0.3 which will lower the saturation magnetization. A similar trend was reported by Asghar et al., who observed a progressive decrease in the saturation magnetization (Ms) from 60.95 emu g⁻¹ (x = 0.0) to 48.84 emu g⁻¹ (x = 0.1) and 24.44 emu g⁻¹ (x = 0.2), despite an increase in crystallite size from 8.71 to 17.44 and 23.24 nm, respectively^58^. The authors attributed this behavior to the substitution of Fe³⁺ ions (5 µB) by Sm³⁺ ions (1 µB) at the octahedral (B) sites. Since the net magnetization in nickel ferrites arises from the difference between the magnetic moments of the B and A sublattices, the incorporation of Sm³⁺ ions, with their lower magnetic moment, reduces the magnetic moment of the B sublattice, leading to a decrease in the saturation magnetization.

**Fig. 8:**
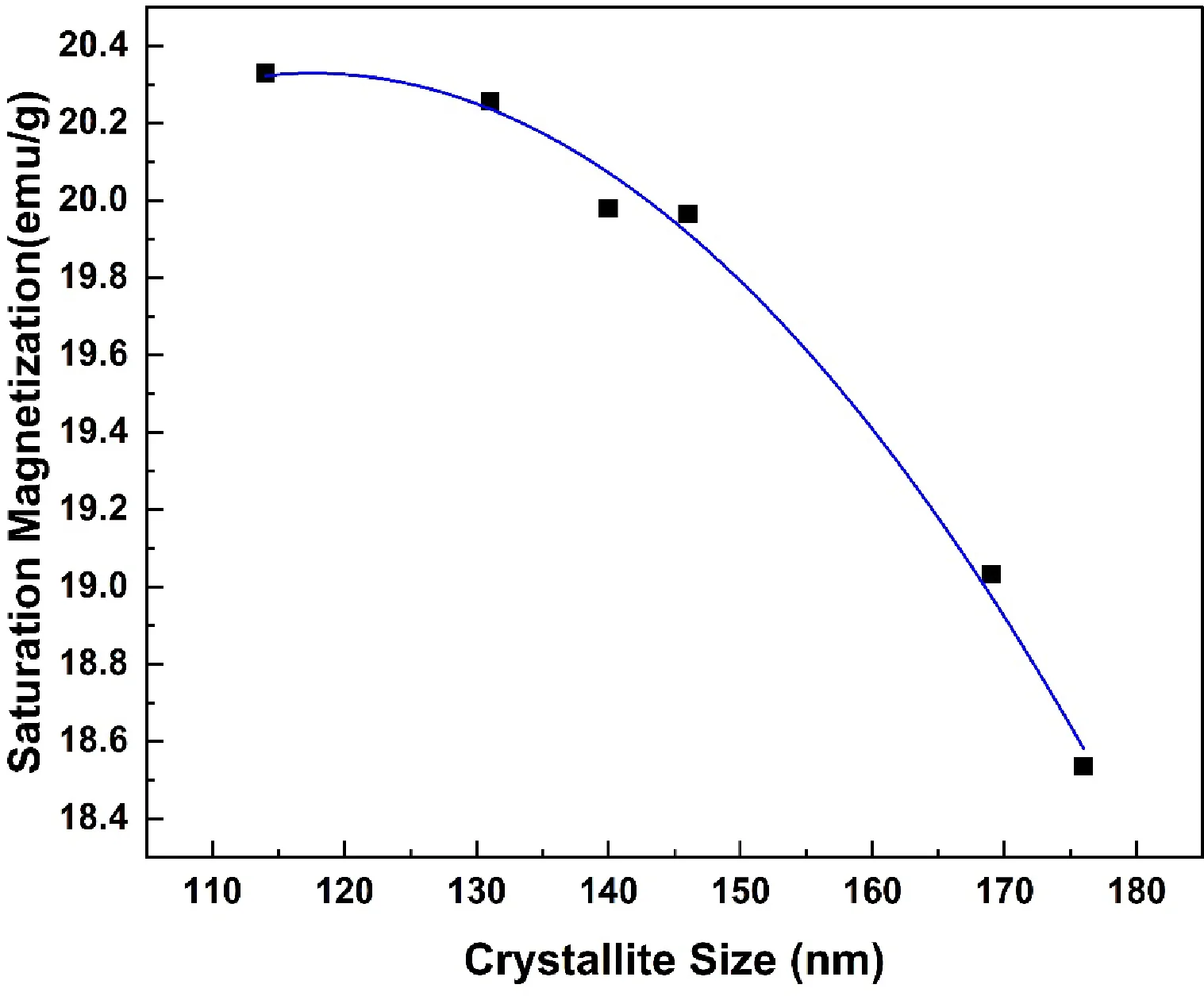
The relation between saturation magnetization and crystallite size of Cu_0.7_Mg_0.3−x_ Ni_x_Fe_1.7_Cr_0.3_O₄ (0.05 ≤ x ≤ 0.30).

Figure 8 illustrates an inverse correlation in the present system. Here, Ms decreases as crystallite size increases, a phenomenon likely driven by Ni²⁺ substitution. This substitution facilitates a cation redistribution that induces magnetic dilution of the octahedral (B) sublattice and subsequently weakens the Fe^+ 3^–O^− 2^-–Fe^+ 3^ superexchange interactions. Because Ni²⁺ inclusion causes Fe³⁺ ions to be redistributed between tetrahedral (A) and octahedral (B) sites and increases Cr³⁺ occupation at B-sites, the saturation magnetization reduces even if the crystallite size increases with Ni substitution. This outweighs the expected positive contribution from greater crystallite size and lowers the net magnetic moment difference (MB-MA). Furthermore, this trend may be attributed to noncollinear spin disorder occurring at or near the nanoparticle surface.

Aruna et al.^37^ investigated Ni_x_Cu₁₋ₓFe₂O₄ (where x ranges from 0.1 to 0.9), synthesized using the sol-gel technique. They reported a decrease in saturation magnetization (Ms) as the Ni content increased. This trend was explained by the two-sublattice model, which includes A-A, A-B, and B-B interactions. Specifically, the presence of Ni at B-sites causes Fe³⁺ ions to migrate to A-sites, weakening the dominant A-B exchange interaction. In contrast, while Aruna et al. observed a significant reduction of 27% in Ms (from 9.002 to 6.595 emu/g) when x increased from 0.1 to 0.3, our samples showed a more moderate decrease of 8% (from 20.258 to 18.537 emu/g) over the same range of substitution. A similar trend was reported by Ali Raza et al^59^. for Ni-substituted cobalt ferrite, where Ms decreased in the 4% and 6% Ni-doped samples compared to pure cobalt ferrite. This reduction was attributed to a combination of cation redistribution, spin canting effects, and increased surface-to-volume ratios in the nanoparticles.

In contrast to the findings of Yang and Wang, where saturation magnetization (Ms) was reported to increase with average crystallite size for Cu₀.₂Mg₀.₃₅Zn₀.₄₅Fe₂O₄^60^. It is evident from Table 3 that the examined samples have low coercivity values, which suggests that they are members of the soft ferritin family. A direct indicator of magnetocrystalline anisotropy that changes with nickel content is coercivity (H_c_). However, the samples under evaluation showed low coercivity values (53.5–44.3 G) with irregular fluctuation, these low values are indicative of their classification as members of the soft ferrite family. This irregularity arises from a complex interplay of competing magnetocrystalline anisotropy factors^51,61^ and variations in grain size near the critical single-domain threshold^62^. Furthermore, domain wall pinning by microstructural defects, coupled with alterations in super-exchange interactions driven by cation redistribution^63^, significantly influences the magnetic response. When combined with stress-induced anisotropy^64^, these concurrent mechanisms drive fluctuations in both the effective anisotropy field and domain wall mobility. Consequently, these factors culminate in the erratic coercivity behavior observed across the Ni substitution range. It is also evident from Table 3 that all samples had squareness ratios below 0.5, which suggests that the samples exhibit single domain particles^34,65^.

The observed magnetic behavior in our Cu_0.7_Mg_0.3-x_Ni_x_Fe_1.7_Cr_0.3_O_4_ system (0.05 ≤ x ≤ 0.30) aligns with established models for soft ferrites, where low coercivity (53.5–44.3 G) and its irregular fluctuations arise from the interplay of magnetocrystalline anisotropy, grain size variations near the single-domain threshold, and domain wall pinning. Furthermore, the 8% decrease in Ms (from 20.258 to 18.537 emu/g) as x increases from 0.1 to 0.3 is attributed to Ni^2+^ substitution at B-sites. This triggers Fe^3+^ migration to A-sites, weakening the A–B super-exchange interactions, a trend consistent with the reported findings. These concurrent factors including stress-induced anisotropy and cation redistribution collectively modulate the effective anisotropy field and domain wall mobility across the Ni substitution range. The measured Ms (18.5–20.3 emu g⁻¹) and Hc (44–57 G) values are lower than those typically required for biomedical applications such as magnetic hyperthermia and MRI contrast agents, but are well suited for soft magnetic and high-frequency electronic applications^66^.

Ni substitution successfully modifies the magnetic characteristics without severely altering the ferrimagnetic ordering, as evidenced by the very slight decrease in saturation magnetisation of about 8% with increasing Ni²⁺ concentration. The compositions’ soft magnetic character is confirmed by the preservation of low coercivity (44–57 G) and just a slight drop in Ms. Therefore, it is not anticipated that the observed magnetic behaviour will negatively impact their performance in high-frequency electronic and soft magnetic applications, where magnetic stability and low hysteresis losses are typically more crucial than reaching the maximum saturation magnetisation.

### Frequency-dependent dielectric response of Ni-rich CMFC ferrites

**Fig. 9:**
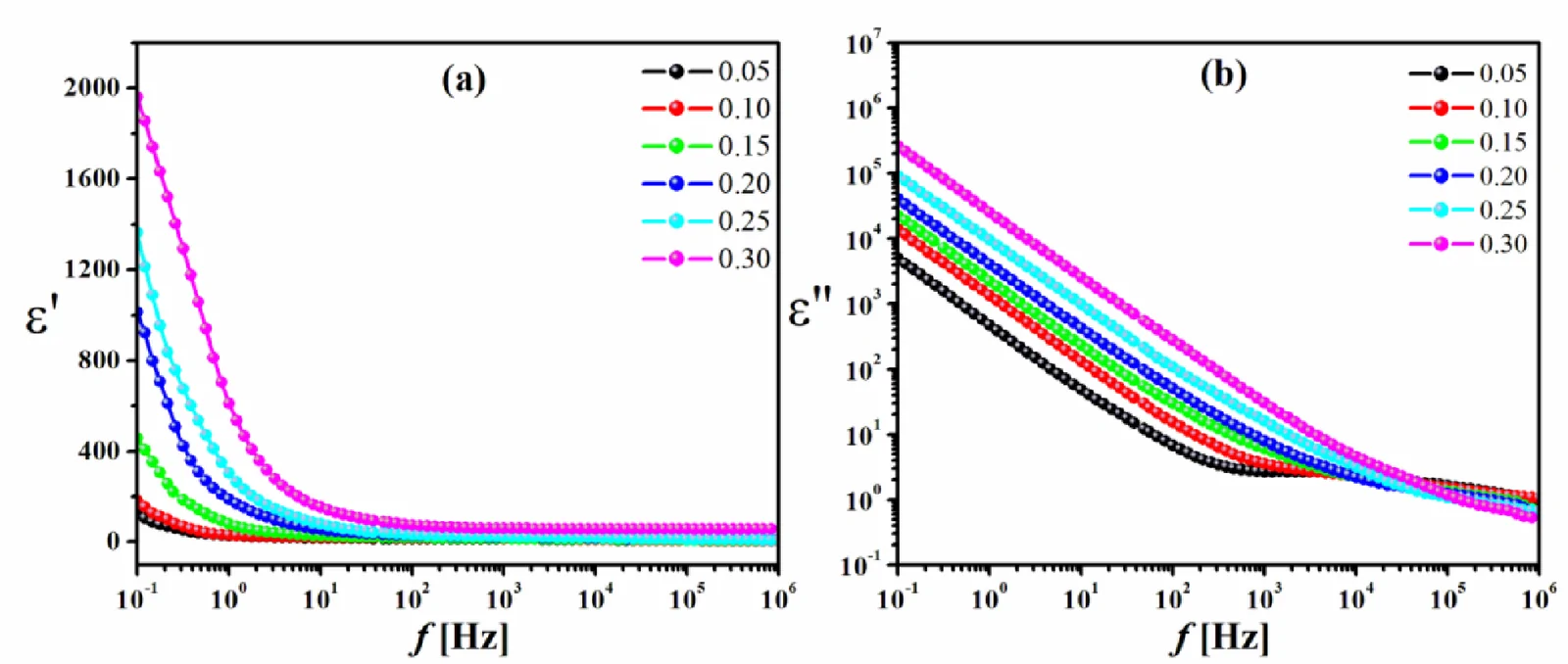
(**a**) Variation of the dielectric constant (ε′), and (**b**) dielectric loss (ε″) with frequency for Cu_0.7_Mg_0.3−*x*_Ni_x_Fe_1.7_Cr_0.3_O₄ compositions (0.05 ≤ *x* ≤ 0.30) at 30 °C.

The frequency-dependent dielectric behavior of the Cu_0.7_Mg_0.3−*x*_Ni_x_Fe_1.7_Cr_0.3_O₄ ferrite system was examined in the range 10⁻¹−10⁶ Hz, and at 30 °C. The dielectric constant ε′, shown in Fig. 9a, exhibits a strong dispersion at low frequencies followed by a gradual decrease as the frequency increases.

Although Maxwell–Wagner interfacial polarization dominates the dielectric response in the investigated frequency range, other polarization mechanisms may also contribute. Electronic polarization is nearly frequency independent, whereas ionic polarization occurs mainly at much higher frequencies and is therefore negligible. Dipolar polarization may provide a minor contribution; however, the dielectric behavior is primarily controlled by interfacial polarization and electron hopping between Fe²⁺/Fe³⁺ and Ni²⁺/Ni³⁺ ions, in agreement with previous studies on spinel ferrites^64,67^.

This behavior is typical of ferromagnetic spinel and can be explained as consisting of well-conducting grains separated by poorly conducting grain boundaries^68–70^. At low frequencies, charge carriers accumulate at the grain boundaries, which possess higher resistivity, leading to significant space-charge polarization and consequently high ε′ values. As the frequency increases, the charge carriers are unable to follow the rapidly alternating electric field, causing the grain boundary contribution to diminish. The dielectric response then becomes dominated by the grains, which exhibit lower polarization, resulting in the observed decrease in ε′. Additionally, Ni substitution plays a crucial role in tuning the dielectric response of the Cu_0.7_Mg_0.3−*x*_Ni_x_Fe_1.7_Cr_0.3_O₄ system. The increase of Ni content enhances the concentration of electrically active cations at the octahedral sites and thereby strengthens the electron hopping between Fe²⁺/Fe³⁺ and Ni²⁺/Ni³⁺ ion pairs, which contributes to increased space-charge polarization^70,71^. This effect becomes particularly pronounced at higher Ni content (*x*= 0.30), where the dielectric constant attains its highest values over the investigated frequency range. In addition to the electronic contribution, the microstructure is also markedly affected by Ni substitution^72^. The compact microstructure at higher Ni concentration facilitates charge accumulation at the interfaces between the relatively conducting grains and the more resistive grain boundaries, reinforcing the Maxwell–Wagner type interfacial polarization, especially at low frequencies.

On the other hand, the dielectric loss ε″, presented in Fig. 9b, also shows strong frequency dependence, with high values at low frequencies that decrease steadily as the frequency increases. At low frequencies, the dielectric response is primarily governed by conduction losses and space charge polarization^73–75^. In this regime, charge carriers are able to follow the slowly varying electric field, leading to enhanced drift motion and energy dissipation^67^. The accumulation of charges at grain boundaries, which act as resistive barriers due to their structurally disordered nature, further amplifies these effects. Such grain boundary polarization, often described by the Maxwell–Wagner model, results in significant dielectric dispersion and loss^75^. As the frequency increases, charge carriers can no longer respond effectively to the alternating field, thereby reducing the influence of space charge and grain boundary effects, and shifting the dielectric behavior toward intrinsic lattice contributions^67,76^. Compared to intermediate compositions, the higher compositions show slightly higher losses at low frequencies, which can be attributed to the increased density of grain boundaries in finer-grained microstructures. Nevertheless, at high frequencies, ε″ values converge, suggesting that grain boundary effects become negligible and the dielectric response is dominated by intrinsic grain conduction.

### Electrical relaxation mechanisms in Ni-rich CMFC ferrites

**Fig. 10:**
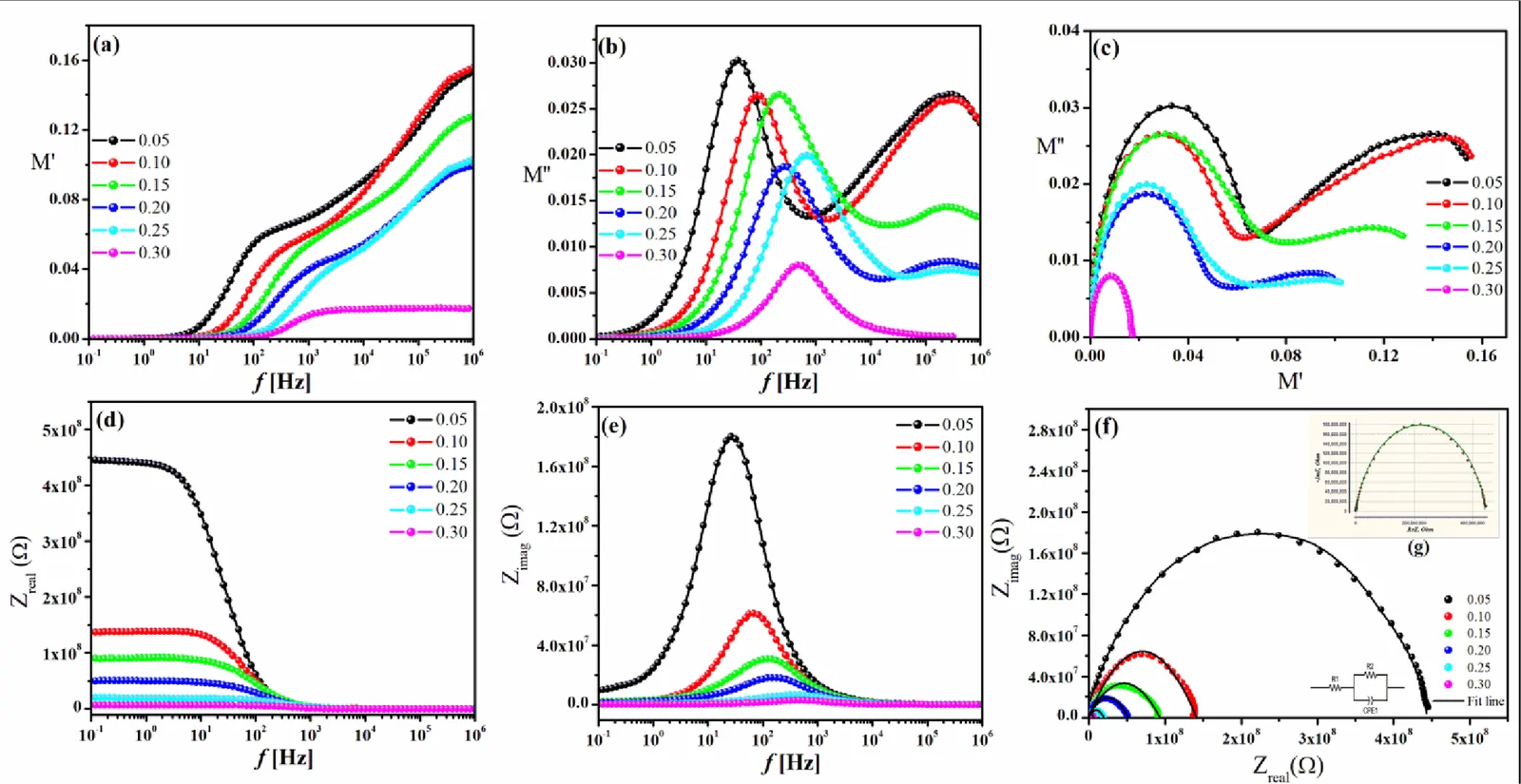
(**a**, **b**) Real and Imaginary part of electric modulus (M′ and M″) versus frequency, (**c**) Nyquist plot (M′ vs. M″), (**d**, **e**) Frequency dependence of Z_real_ parameter and *Z*_imag_ parameter for Cu_0.7_Mg_0.3−*x*_Ni_x_Fe_1.7_Cr_0.3_O₄ compositions (0.05 ≤ *x* ≤ 0.30) at 30 °C, and (**f**) Nyquist plot for (Z_imag_ vs. Z_real_). The experimental impedance data were fitted using the *EIS Spectrum Analyzer* software (www.abc.chemistry.bsu) based on the R₁ + (R₂‖CPE) equivalent circuit model. The inset (**g**) shows the Nyquist plot for *x* = 0.05, highlighting the fitted semicircle obtained by the same software.

Figure 10a, shows the real part of the modulus (M′) of Cu_0.7_Mg_0.3−*x*_Ni_x_Fe_1.7_Cr_0.3_O₄ compositions. M′ remains close to zero at low frequencies due to electrode polarization and grain boundary resistance^68,69^. With increasing frequency, M′ rises gradually, reflecting the activation of short-range relaxation processes within the grains^77^. The samples with the highest x-composition display the lowest values of the real part of the electric modulus (M′) throughout the entire measured frequency range. This result indicates that Ni substitution enhances charge carrier mobility and reduces grain boundary effects. The improved hopping conduction between Fe²⁺/Fe³⁺ and Ni²⁺/Ni³⁺ ions at octahedral sites accounts for this enhanced conductivity^78^. Figure 10b presents the imaginary part of the modulus (M″), which displays a relaxation peak for each composition. These peaks shift toward higher frequencies with increasing Ni content, confirming non-Debye type relaxation behavior typical of ferrites with heterogeneous microstructures^79^. The higher Ni-rich compositions show relatively weaker peak intensity, consistent with its higher conductivity: when carriers move more freely, the relaxation peak is suppressed because less energy is stored and released during polarization^80,81^.

Overall, the electric modulus analysis demonstrates that dielectric relaxation in these ferrites is governed by the interaction between grain interiors and grain boundaries. Ni substitution modifies this balance, with intermediate compositions (x ≈ 0.15–0.25) showing stronger relaxation peaks and higher M′ values, while the higher Ni-composition exhibits the lowest M′ and suppressed M″ peak, confirming its highest conductivity and reduced interfacial polarization.

The plots of the complex electric modulus representation (M′ vs. M″) in Fig. 10c, exhibit distorted semicircular arcs rather than ideal Debye-type semicircles, confirming the presence of distributed relaxation times and non-Debye behavior typical of polycrystalline ferrites^69^. This distortion arises from microstructural heterogeneity.

Obviously, the arc positions and shapes are strongly dependent on Ni substitution. Compositions with lower Ni content (*x* = 0.05–0.15) show broader arcs with higher M′ and M″ values, reflecting slower relaxation processes and stronger grain boundary impedance. These samples store and dissipate more energy, consistent with limited hopping conduction and higher resistive interfaces. Intermediate compositions (*x* = 0.20–0.25) exhibit moderately compressed arcs, indicating improved relaxation dynamics and reduced interfacial resistance, representing a transitional regime where grain conductivity begins to dominate over boundary effects. The highest Ni- composition (*x*= 0.30) lies closest to the origin, exhibiting the lowest M′ and M″ values. Since lower M′ corresponds to higher conductivity, this result confirms that higher Ni substitution enhances charge carrier mobility and reduces grain boundary impedance. In addition, the suppressed modulus response indicates rapid carrier response, consistent with enhanced hopping conduction between Fe²⁺/Fe³⁺ and Ni²⁺/Ni³⁺ ions at octahedral sites^77,78^. The reduced M″ peak intensity further supports this conclusion, as higher conductivity diminishes the extent of relaxation-related energy dissipation.

### Impedance spectroscopy

Figure 10d illustrates the spectra of the real component of impedance (Z′). The frequency exhibits significant fluctuations across all compositions. At low frequencies, the impedance values are rising, indicating that the grain boundaries impede charge movement. As the frequency increases, Z′ rapidly decreases, approaching near zero in the high-frequency the frequency range. Z’ progressively diminishes as the concentration of Ni²⁺ increases (x → 0.30). This indicates that the material exhibits increased electrical conductivity. Ni²⁺ assumes the role of Mg²⁺, influencing the distribution of cations and facilitating the mobility of Fe²⁺/Fe³⁺. Comparable decreases in grain boundary resistance with the incorporation of Ni have been seen in Ni–Mg ferrite systems^82,83^. The spectra of the imaginary component of impedance (Z″) in Fig. 10e exhibit peaks of relaxation characteristic of dielectric relaxation phenomena. The relaxation peaks diminish in size and shift to higher frequencies with increasing nickel content. This pattern indicates that the charge carriers exhibit increased velocity and experience diminished polarization loss. The variation in relaxation frequency aligns with enhanced hopping conduction, as Ni²⁺ ions promote electron transfer between Fe ions at octahedral locations. Comparable peak shifts have been seen in Ni–Cu ferrites, wherein Ni substitution enhances dielectric relaxation^84^.

In order to clarify the conduction mechanism in the analyzed samples and emphasize the effect of NI doping at different concentrations, experimental data is presented as a Nyquist plot in Fig. 10f. The Nyquist plots of the investigated ferrite compositions exhibit unique semicircular arcs, signifying grain boundary-controlled conduction in spinel ferrites. At a low Ni content (x = 0.05), the semicircle diameter is significant indicating a high grain boundary resistance and reduced charge mobility. As the concentration of Ni²⁺ increases, the diameter of the semicircle decreases systematically. This indicates that resistance decreases while electrical conductivity increases. The semicircles are similarly noted to be positioned lower, with their centers situated below the Zreal axis. These characteristics indicate the presence of non-ideal Debye behavior^85^. This pattern aligns with the substitution of Mg²⁺ with Ni²⁺, altering the cation distribution and facilitating electron hopping between Fe²⁺ and Fe³⁺ at octahedral locations. The reduction in arc radius with Ni doping indicates that bulk conduction is increasingly significant compared to grain boundary effects in charge transport. The migration of the semicircle centers towards lower Z′ values further corroborate the notion that charge carrier dynamics are improving. In Ni–Mg–Cu ferrites, the incorporation of Ni reduces the height of the grain boundary barrier and accelerates relaxation processes^83^.

Additionally, the impedance spectra in Fig. 10f of Cu₀.₇Mg₀.₃₋ₓNiₓFe₁.₇Cr₀.₃O₄ (0.05 ≤ x ≤ 0.30) were fitted using *EIS Spectrum Analyzer*software^86,87^. The samples were fitted based on the equivalent circuit model R₁ + (R₂‖CPE)^88^, given by Eq. (9):

9 $$\:Z\left(\omega\:\right)=R1+\frac{1}{\frac{1}{R2}+Q.{\left(j\omega\:\right)}^{n}}$$

where R₁ denotes the series resistance related to bulk and grain boundary conduction, R₂ signifies the parallel resistance linked to charge transfer or interfacial polarization, Q represents the constant phase element (CPE) perfector indicative of non-ideal capacitance, and n is the CPE exponent (0 ≤ *n* ≤ 1) that characterizes the deviation from ideal capacitive behavior. The angular frequency is defined as ω = 2πf. At low nickel concentration (x = 0.05–0.10), R₁ is comparatively elevated (4.5 × 10⁵–9.09 × 10⁵ Ω), but R₂ attains values in the 10⁸ Ω range as shown in Table 4, signifying that grain boundaries predominantly influence the impedance response. The low Q values and moderate n (~ 0.86–0.89) indicate restricted capacitive storage with almost optimal polarization.

At intermediate nickel concentrations (x = 0.15–0.25), R₁ stabilizes around 3.96–3.97 kΩ, indicating enhanced grain conduction. R₂ diminishes to approximately 10⁷ Ω, aligning with reduced grain boundary impedance. The Q values rise (up to 1.27 × 10⁻¹⁰), whereas n decreases to approximately 0.79–0.80, signifying enhanced non-ideal capacitive behavior attributed to microstructural variability. This domain is characterized by increased electron transfer between Fe²⁺/Fe³⁺ and Ni²⁺/Ni³⁺ ions, as evidenced by dielectric studies. At the maximum nickel concentration (x = 0.30), R₁ remains approximately 3.96 kΩ, while R₂ further diminishes to 6.55 × 10⁶ Ω, confirming improved charge diffusion between grains. The Q value is maximized at 1.29 × 10⁻¹⁰, and n increases to 0.90, indicating an improvement towards a more optimal capacitive response. The results correspond with the electric modulus analysis, indicating that Ni-rich compositions had diminished relaxation peaks and enhanced conductivity. These findings are consistent with the electric modulus study, which concluded that compositions rich in Ni reduced relaxation peaks and increased conductivity.

In summary, our assessment of high-frequency (MHz region) suitability was based on indirect evidence from dielectric and impedance analyses, specifically the suppression of dielectric loss and the stabilization of the electric modulus at higher Ni concentrations. These trends indicate reduced interfacial polarization and faster relaxation dynamics, which are standard indicators of potential for high-frequency performance in ferrites. Overall, dielectric and impedance analyses reveal a marked reduction in grain boundary resistance and stable relaxation behavior at elevated frequencies. These findings highlight the promise of the material for extended MHz-frequency applications. Nevertheless, direct measurements within the microwave region are essential, and will be prioritized in future studies to confirm suitability for practical device integration.

Since the electrical characterization was limited to 1 MHz, the proposed suitability for high-frequency electronic applications is restricted to the investigated MHz range. Further microwave-frequency measurements are required to confirm the material’s performance beyond this range.

### Electrical conductivity

**Fig. 11:**
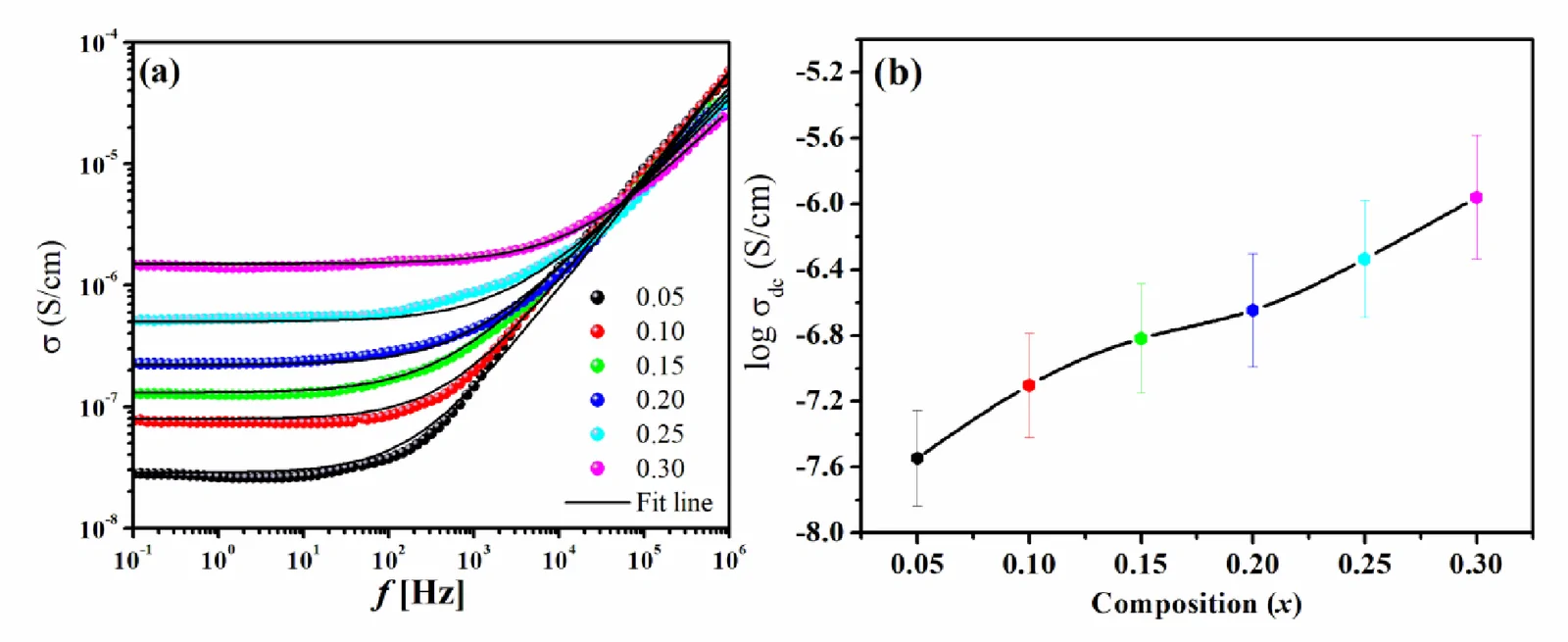
(**a**) AC conductivity versus frequency, and (b) Logarithmic DC conductivity (log σ_dc_) versus composition (*x*) for Cu_0.7_Mg_0.3−*x*_Ni_x_Fe_1.7_Cr_0.3_O₄ compositions (0.05 ≤ *x* ≤ 0.30) at 30 °C.

The electrical conductivity of Cu_0.7_Mg_0.3−*x*_Ni_x_Fe_1.7_Cr_0.3_O_4_ ferrites was investigated as a function of both frequency and Ni substitution level. Figure 11a presents the variation of AC conductivity (σ) with frequency for Ni compositions ranging from x = 0.05 to x = 0.30, while Fig. 11b shows the logarithmic trend of DC conductivity (log σ_dc_) as a function of Ni- doping. It is clear that, the conductivity curves in Fig. 11a at low frequencies, exhibit a plateau region corresponding to the DC conductivity. This behavior is attributed to long-range charge carrier motion, which is limited by grain boundary resistance and interfacial polarization effects^79,80^. As the frequency increases, the conductivity rises steadily, indicating the onset of short-range hopping conduction. This frequency-dependent behavior conforms to the universal power law:

10 $$\sigma _{{{\mathrm{ac}}}} = {\text{ }}\sigma _{{{\mathrm{dc}}}} + {\text{ A}}\omega ^{n}$$

where σ_ac_ denotes the frequency-dependent conductivity assessed under an alternating current field, σ_dc_ denotes the direct current conductivity, A is a temperature- and composition-dependent constant, and *n* is the frequency exponent (0 < *n* < 1), characteristic of disordered systems with localized charge carriers, and ω (= 2πf) denotes angular frequency^80^. The calculated frequency exponent “*n*” was found to lie in the range 0.89 ≤ *n* ≤ 0.71. Such values, being less than unity, are characteristic of disordered systems. However, the observed decrease in “*n”*with increasing Ni doping suggests that, the AC conductivity is controlled by hopping processes^89^.

In addition, the observed increase in conductivity with frequency (Fig. 11a) is attributed to the enhanced hopping of electrons between Fe²⁺/Fe³⁺ and Ni²⁺/Ni³⁺ ions at octahedral sites. Ni substitution modifies the cation distribution and reduces grain boundary impedance, thereby facilitating carrier mobility. The conductivity magnitude increases systematically with Ni content, confirming that higher Ni levels promote more efficient charge transport^90–92^.

Figure 11b further supports this interpretation, showing a monotonic increase in log σ_dc_ with increasing Ni concentration indicating that Ni incorporation significantly enhances the electrical performance of the ferrite system. However, the conductivity analysis reveals that both AC and DC conductivity are strongly influenced by frequency and Ni content. The Ni rich compositions demonstrate the highest conductivity, making them a promising candidate for applications requiring efficient charge transport particularly in high-frequency electronic components.

As previously revealed, grain boundary polarization reduces, and the dielectric response is principally influenced by bulk grain contributions at higher frequencies. The stabilization of ε′ is further demonstrated by Ni-induced hopping mechanisms that preserve conductivity in the MHz range. Also, the increase of crystallite sizes lowers defect-induced relaxation, and electric modulus analysis indicates non-Debye behavior characterized by reduced relaxation peaks in the nickel-rich samples. These mechanisms facilitate a uniform dielectric performance and improve AC/DC conductivity, making Ni-rich CMFC ferrites viable options for MHz frequency range applications.

## Conclusion

In this study, the effect of Ni substitution on the structural, optical, magnetic, and dielectric properties of Cu₀.₇Mg₀.₃₋ₓNiₓFe₁.₇Cr₀.₃O₄ spinel ferrites were systematically investigated. XRD analysis confirmed the formation of a single-phase cubic spinel structure with Fd–3 m symmetry for all compositions, indicating the structural stability of the multi-cation ferrite system. The observed increase in crystallite size and optical band gap with increasing Ni concentration reflects the strong influence of cation redistribution and lattice modification on the electronic structure, while the gradual decrease in refractive index suggests reduced ionic polarizability within the lattice. Magnetic measurements revealed soft ferrimagnetic behavior throughout the compositional series. Although the Ni content increased significantly, the saturation magnetization decreased only slightly, whereas the low coercivity values (44–57 G) confirmed the preservation of magnetic softness. The dielectric behavior was predominantly governed by Maxwell–Wagner interfacial polarization, and the non-Debye relaxation characteristics confirmed the heterogeneous nature of the system. Both AC and DC conductivities increased systematically with Ni substitution, which can be attributed to enhanced electron hopping between Fe²⁺/Fe³⁺ and Ni²⁺/Ni³⁺ ions together with reduced grain boundary resistance. The obtained correlations highlight the potential of Ni-substituted ferrites for electronic applications within the investigated MHz frequency range. However, further microwave-frequency characterization is required to validate their performance beyond 1 MHz. Although further investigations over wider frequency and temperature ranges are desirable to fully evaluate their operational performance, the present results indicate that Ni-rich compositions offer improved dielectric stability and electrical conductivity while maintaining favourable soft magnetic characteristics. Overall, this study provides the potential of the Ni-rich ferrites with deeper insight into cation-engineered spinel ferrites and establishes a useful foundation for the development of multifunctional ceramic materials for magnetic data storage and other high-frequency electronic applications. Future studies will extend the measurements to the microwave region to further evaluate the applicability of these ferrites in practical high-frequency devices.

**Table 1:** Crystallite Size values, and dislocation density of Cu_0.7_Mg_0.3−x_Ni_x_Fe_1.7_Cr_0.3_O_4_, 0.05 ≤ x ≤ 0.3.

Ni	0.05	0.1	0.15	0.2	0.25	0.3
Crystallite size (nm)	114	131	140	146	169	176
δ	0.00877	0.00763	0.00714	0.00685	0.00592	0.00568
E_g_ (eV)	3.22	3.37	3.50	3.58	3.60	3.72

**Table 2:** FTIR bands comparative between our work and others.

Band	Our work	Reference^37^	Reference^38^	Reference^39^
Higher frequency ν1	565.36–576.25 cm^− 1^	566 cm^− 1^	600 and 500 cm^− 1^	569.92 cm^− 1^.
lower frequency ν2	421.337–439.79 cm^− 1^	438 cm^− 1^	Close to 400 cm^− 1^	490 cm^− 1^

**Table 3:** Magnetic properties of Cu_0.7_Mg_0.3−x_Ni_x_Fe_1.7_Cr_0.3_O_4_, 0.05 ≤ x ≤ 0.3.

Ni content, x	Saturation Magnetization(emu/g)	Coercivity Hc(G)	Remanent magnetization (emu/g)	Squareness
0.05	20.330	53.507	0.9835	48.378 × 10^− 3^
0.1	20.258	45.602	0.83694	41.316 × 10^− 3^
0.15	19.980	55.578	1.0404	52.072 × 10^− 3^
0.2	19.966	57.407	1.1081	55.503 × 10^− 3^
0.25	19.033	44.386	0.77934	40.946 × 10^− 3^
0.3	18.537	51.900	0.91687	49.463 × 10^− 3^

**Table 4:** Fitted impedance parameters for Cu₀.₇Mg₀.₃₋ₓNiₓFe₁.₇Cr₀.₃O₄ ferrites based on the R₁ + (R₂‖CPE) equivalent circuit.

Ni content (x)	*R*₁ (Ω)	*R*₂ (Ω)	Q (F·sⁿ⁻¹)	*n*	Error%
0.05	4.50 × 10⁵	4.44 × 10⁸	2.72 × 10⁻¹¹	0.860	0.364
0.10	9.09 × 10⁵	1.39 × 10⁸	3.18 × 10⁻¹¹	0.890	0.245
0.15	3970.5	9.05 × 10⁷	5.94 × 10⁻¹¹	0.803	1.183
0.20	3970.4	5.05 × 10⁷	9.55 × 10⁻¹¹	0.800	0.470
0.25	3960.6	1.95 × 10⁷	1.27 × 10⁻¹⁰	0.790	0.897
0.25	3960.6	1.95 × 10⁷	1.27 × 10⁻¹⁰	0.791	0.368
0.30	3960.5	6.55 × 10⁶	1.29 × 10⁻¹⁰	0.901	0.369

## Data Availability

The data that support the findings of this study are available from the corresponding author upon reasonable request.
